# Characterization of the mitochondrial genomes of two toads, *Anaxyrus americanus* (Anura: Bufonidae) and *Bufotes pewzowi* (Anura: Bufonidae), with phylogenetic and selection pressure analyses

**DOI:** 10.7717/peerj.8901

**Published:** 2020-04-14

**Authors:** Yu-Ting Cai, Qin Li, Jia-Yong Zhang, Kenneth B. Storey, Dan-Na Yu

**Affiliations:** 1 College of Chemistry and Life Science, Zhejiang Normal University, Jinhua, Zhejiang Province, China; 2 Key Lab of Wildlife Biotechnology, Conservation and Utilization of Zhejiang Province, Zhejiang Normal University, Jinhua, Zhejiang, China; 3 Department of Biology, Carleton University, Ottawa, Ontario, Canada

**Keywords:** Bufonidae, Mitochondrial genomes, Phylogenetic relationships, Positive selection

## Abstract

Mitogenomes are useful in analyzing phylogenetic relationships and also appear to influence energy metabolism, thermoregulation and osmoregulation. Much evidence has accumulated for positive selection acting on mitochondrial genes associated with environmental adaptation. Hence, the mitogenome is a likely target for environmental selection. The family Bufonidae (true toads) has only nine complete and four partial mitogenomes published compared to the 610 known species of this family. More mitogenomes are needed in order to obtain a clearer understanding of the phylogenetic relationships within Bufonidae that are currently controversial. To date, no mitogenomes have been reported from the genera *Anaxyrus* and *Bufotes*. *Anaxyrus americanus* can live in low temperature environments and *Bufotes pewzowi* can live in high salinity environments. We sequenced the mitogenomes of these two species to discuss the phylogenetic relationships within Bufonidae and the selection pressures experienced by specimens living in low temperature or saline environments. Like other toads, the circular mitogenomes of both species contained the typical 37 genes. *Anaxyrus americanus* had the highest A+T content of the complete mitogenome among the Bufonidae. In addition, *A. americanus* showed a negative AT-skew in the control region, whereas *Bufotes pewzowi* showed a positive AT-skew. Additionally, both toad species had unique molecular features in common: an *ND1* gene that uses TTG as the start codon, an extra unpaired adenine (A) in the anticodon arm of *trnS* (AGY), and the loss of the DHU loop in *trnC*. The monophyly of Bufonidae was corroborated by both BI and ML trees. An analysis of selective pressure based on the 13 protein coding genes was conducted using the EasyCodeML program. In the branch model analysis, we found two branches of *A. americanus* and *Bufotes pewzowi* that were under negative selection. Additionally, we found two positively selected sites (at positions 115 and 119, BEB value > 0.90) in the ND6 protein in the site model analysis. The residue D (119) was located only in *A. americanus* and may be related to adaptive evolution in low temperature environments. However, there was no evidence of a positively selected site in *Bufotes pewzowi* in this study.

## Introduction

Bufonidae, the true toads, consists of 33 genera and 610 species (according to the Amphibian Species of the World 6.0, an online reference (https://amphibiansoftheworld.amnh.org/); 16 Apr. 2019) (Frost, 2014) and is one of the most species-rich amphibian families (Frost et al., 2006; Pramuk, 2006). However, there are only nine complete mitochondrial genomes (mitogenomes) of Bufonidae species published in GenBank along with four partial genomes (Cao et al., 2006; Dong & Yang, 2016; Igawa et al., 2008; Jacob Machado et al., 2018; Machado, Lyra & Grant, 2016; Jiang et al., 2017b; Wang et al., 2013; Yang et al., 2016; Zhang et al., 2005; Zhang et al., 2013; Zhang et al., 2016). These molecular data are not sufficient to represent the biodiversity of Bufonidae species. Pauly, Hillis & Cannatella (2004), Frost et al. (2006) and Van Bocxlaer et al. (2009) corroborated the monophyly of Bufonidae by using partial mitochondrial DNA sequences. Portik & Papenfuss (2015) also confirmed the monophyly of Bufonidae, based on 243 taxa and 13 loci including nine nuclear genes and four mitochondrial genes. Furthermore, the phylogenetic relationships within Bufonidae remain controversial (Graybeal, 1997). As for intergeneric relationships, the genus *Bufo* was strongly confirmed as monophyletic (Graybeal, 1997; Dong & Yang, 2016). On the contrary, the polyphyly of *Bufo* was recovered in several other studies (Maxson, 1984; Pyron & Wiens, 2011; Brandvain et al., 2014; Jiang et al., 2018) and the paraphyly of South American toads was also supported (Frost et al., 2006; Pramuk, 2006).

Mitogenomes of Anura are closed, double-stranded circular molecules of about 16-24 kb in length that include 2 ribosomal RNA genes (12S and 16S rRNA), 22 transfer RNA genes (tRNAs), 13 protein-coding genes (PCGs), and one control region (CR; also known as the D-loop region) (Kakehashi et al., 2013; Cai et al., 2019). Mitogenomes (synonym mitochondrial genomes) are useful molecular markers for analyzing population structure, phylogenetic relationships and divergence time (Igawa et al., 2008; Masta et al., 2002) due to their small size, lack of recombination, rapid evolution rate, conserved gene content and genomic organization, and maternal inheritance (Masta et al., 2002; Zhang et al., 2003). In addition, mitogenomes have high substitution rates and these substitutions may have relevant effects on fitness and metabolism (Carapelli et al., 2019). Although mitogenomes are usually thought to be under neutral or nearly neutral selection, evidence has accumulated for positive selection acting on mitochondrial genes associated with environmental adaptations (Carapelli et al., 2019; Shen et al., 2010). Hence, the mitogenome is a likely target for environmental selection and it is useful in analyzing positive selection or natural selection (Zhou et al., 2014).

Mitochondria are called the powerhouses of the cell because they synthesize most of the ATP necessary to drive cell functions (McBride, Neuspiel & Wasiak, 2006). Numerous environmental factors can affect the growth and survival of amphibians, including temperature and salinity (Yaghobi et al., 2018). Temperature has a great effect on the bioenergetic demands and metabolic adaptation of ectotherms (Sun et al., 2018a). For example, negative selection was found in the mitochondrial protein-coding genes of *Glandirana* (Xia et al., 2014). Malyarchuk et al. (2010) postulated that the amino acid changes in cytochrome B (*CYTB*) might be advantageous in cold climatic conditions and make it possible for several Siberian salamanders to live in extreme cold environments. Adaptation to saline environments probably needs more energy devoted to osmoregulation (Xia et al., 2017) and several genes in the mitogenome appear to be under positive selection for the role that they play in energy metabolism (Caballero et al., 2015). Salinity and osmoregulation are also significant factors in the survival and fitness of all amphibians because their skin is highly water-permeable (Yaghobi et al., 2018). Indeed, Whitehead (2009) found that salinity differences were associated with amino acid changes in the mitochondrial protein-coding genes of *Fundulus* fish populations along the Atlantic coast.

Mitogenomes could have an impact on energy metabolism, thermoregulation and osmoregulation. However, few studies of Anura have examined the potential involvement of mitogenome adaptations to life in extreme environments. *Anaxyrus americanus* (synonym *Bufo americanus*) is a small American toad (Frost et al., 2006; Masta et al., 2002). It is widely distributed across North America (Haislip et al., 2011) and can live in places with low nighttime temperatures below 0 °C for as much as eight months of the year. However, contrary to various frog species in the same environment that endure whole body freezing in winter (Storey & Storey, 2017), *A. americanus* is freeze intolerant (Bergstrom, 2010; Storey & Storey, 1986) and typically overwinters by digging underground to below the frostline. *Bufotes pewzowi* (synonym *Bufo pewzowi*) is distributed in China, Kazakhstan, Kyrgyzstan, Mongolia and Uzbekistan; belonging to the *Bufo viridis* group, the species can withstand low temperatures and a high level of water salinity (Katz et al., 1981; Ren et al., 2009). Hence, we hypothesized that the mitogenome adaptations of these two species that live in low temperature (*A. americanus*) or low temperature and high water salinity (*Bufotes pewzowi*) environments may be affected by these extreme environments. Therefore, in the present study, we sequenced and annotated the complete mitogenomes of *A. americanus* and *Bufotes pewzowi*, these being the first reported mitogenomes for the genera *Anaxyrus* and *Bufotes*, and clarified their mitogenome differences and similarities in comparison with other Bufonidae species. We also performed evolutionary relationship analyses to discuss the intergeneric relationships among the Bufonidae and evaluated potential positive selection in *A. americanus* and *Bufotes pewzowi* by using the branch model and the site model. These two complete mitogenomes represent two genera for which complete mitogenomes were previously unknown. Hence, our results not only supplement the limited molecular data previously available for Bufonidae, but also examine the monophyly of Bufonidae and explore the idea of adaptive evolution of mitogenomes in response to extreme environmental stresses.

## Materials and Methods

### Sample Collection and DNA extraction

The specimens of *A. americanus* and *Bufotes pewzowi* were collected in Ottawa, Ontario, Canada (45°25.38′N, 75°43.11′W) and Aksu, Xinjiang, China (41°10.54′N, 80°16.81′E), respectively. Both were identified by J.Y. Zhang. Toe-clip samples of both species were stored at −80 °C in the Institute of Ecology, Zhejiang Normal University. Our experimental procedures complied with the current regulations on animal welfare and research in China and Canada. The Animal Research Ethics Committees of Zhejiang Normal University and Carleton University approved the experimental design (No.1082196).

### PCR amplification, and sequencing

Total DNA was extracted from the toe-clips samples of *A. americanus* or *Bufotes pewzowi* using an Ezup Column Animal Genomic DNA Purification Kit (Sangon Biotech Company, Shanghai, China). The 11 universal primers for standard polymerase chain reaction (PCR) amplification of mitogenomes were slightly modified according to Yu et al. (2015) and Zhang et al. (2013) and 15 specific primers were designed based on the sequenced fragments from universal primers using Primer Premier 5.0 (PREMIER Biosoft International, CA, USA) (Tables S1 and S2). All PCR amplifications were carried out in a 50 µL reaction mixture and the procedures were performed using an Eppendorf Thermal Cycler (Mastercycle® nexus GSX1, Hamburg, Germany). We used both standard PCR and Long-PCR methods with *TaKaRa Ex-Taq* and *TaKaRa LA-Taq Kits* (Takara Biomedical, Dalian, China). These two methods were slightly modified from Yu et al. (2015) and Zhou et al. (2009). All PCR products were detected by electrophoresis on 1% agarose gels, and sequences were obtained in an automated DNA sequencer (ABI 3730) by Sangon Biotech Company (Shanghai, China).

### Mitogenome annotation and sequence analyses

Sequences were checked and assembled using SeqMan (Lasergene version 5.0) (Burland, 2000). The 22 tRNAs were identified by their cloverleaf secondary structure using tRNAscan SE 1.21 (Lowe, 1997) (http://lowelab.ucsc.edu/tRNAscan-SE/) or determined by comparison with the available tRNA genes of closely related anurans downloaded from GenBank. Location of the 13 PCGs and 2 rRNA genes were determined by comparison with homologous sequences of mtDNA from other Bufonidae species using ClustalW (Thompson, Higgins & Gibson, 1994) and then PCGs were checked and translated to amino acids using the vertebrate mitogenome code by Mega 5.0 (Tamura et al., 2011). The mitogenome maps of *A. americanus* and *Bufotes pewzowi* were constructed using GenomeVx (http://wolfe.gen.tcd.ie/GenomeVx/) (Conant & Wolfe, 2008). The A+T and C+G content values, codon usage and relative synonymous codon usage (RSCU) of protein-coding genes were calculated using Mega 5.0 (Tamura et al., 2011). Nucleotide sequence skewness was calculated according to the following formulae: AT-skew = (A −T)/(A +T) and GC-skew = (G −C)/(G +C) (Perna & Kocher, 1995).

### Phylogenetic analyses

To confirm the phylogenetic relationships among Bufonidae, 19 sequences of complete or partial mitochondrial genomes were used. The data set was inclusive of the ingroups of the 2 species from this study, 13 other species from Bufonidae (Cao et al., 2006; Dong & Yang, 2016; Igawa et al., 2008; Jacob Machado et al., 2018; Jiang et al., 2017b; Machado, Lyra & Grant, 2016; Wang et al., 2013; Yang et al., 2016; Zhang et al., 2005; Zhang et al., 2013) and the outgroups of 4 species from *Mannophryne* and *Dendrobatidae* (Lyra et al., 2017; Zhang et al., 2013). Accession numbers of all mitogenomes are summarized in Table 1. The amino acid and nucleotide sequences of the 13 protein-coding genes from all 19 species were employed to construct BI and ML phylogenetic trees according to the methods of Zhang et al. (2018) and Zhou et al. (2009). All of the 13 PCGs were aligned using Clustal W in Mega 5.0 (Tamura et al., 2011) and were analyzed with Gblocks 0.91b (Castresana, 2000) using default settings to select conserved regions. The best partition scheme and evolutionary model were determined with the PartitionFinder v.1.1.1 program (Lanfear et al., 2012), using the Bayesian Information Criterion (BIC) (Schwarz, 1978). For the ML analysis, we used the RAxML program (Stamatakis, 2014) under the GTRGAMMAI model with 1,000 bootstrap replications. For BI analysis, we used MrBayes 3.1.2 (Huelsenbeck & Ronquist, 2001) under the GTR+I+G model. Markov Chain Monte Carlo (MCMC) was run with four chains for 10 million generations, with sampling every 1,000 generations. The first 25% of generations were removed as burn-in, which was decided by checking convergences of -log likelihood (*-lnL*). After the average standard deviation of split frequencies in Bayesian was below 0.01, we judged that the Bayesian analysis had reached sufficient convergence.

**Table 1:** GenBank accession numbers of the species used in constructing the phylogenetic trees.

Family	Genus	Species	GenBank accession number	References
Bufonidae	*Anaxyrus*	*Anaxyrus americanus*	MK855099	
		*Bufo*	*Bufo japonicus*	AB303363.1	Igawa et al. (2008)
				*Bufo gargarizans*	DQ275350.1	Cao et al. (2006)
				*Bufo tibetanus*	JX878885.1	Wang et al. (2013)
				*Bufo stejnegeri*	KR136211.1	Dong & Yang (2016)
				*Bufo gargarizans minshanicus*	KM587710.1	Yang et al. (2016)
			*Bufo gargarizans*	KU321581.1	Jiang et al. (2017a) and Jiang et al. (2017b)
		*Bufotes*	*Bufotes pewzowi*	MK855100	
		*Duttaphrynus*	*Bufo melanostictus*	AY458592.1	Zhang et al. (2005)
		*Leptophryne*	*Leptophryne borbonica*	JX564876.1	Zhang et al. (2013)
		*Melanophryniscus*	*Melanophryniscus moreirae*	KY962391.1	Jacob Machado et al. (2018)
			*Melanophryniscus simplex*	KT221611.1	Machado, Lyra & Grant (2016)
	*Rhinella*	*Rhinella sp.*	KT221613.1	Machado, Lyra & Grant (2016)
Aromobatidae	*Mannophryne*	*Mannophryne trinitatis*	JX564878.1	Zhang et al. (2013)
Dendrobatidae	*Dendrobates*	*Dendrobates tinctorius*	MF069441.1	Lyra et al. (2017)
				*Dendrobates leucomelas*	MF069436.1	Lyra et al. (2017)
		*Dendrobates auratus*	MF069434.1	Lyra et al. (2017)

### Analysis of positive selection

The program EasyCodeML (Gao et al., 2019) was used to analyze the selective pressure on mitogenomes; this is an interactive visual tool for detecting selection in a molecular evolutionary analysis based on CodeML (Yang, 2007). The *ω* ratio is the rate of nonsynonymous (*dN*) versus synonymous (*dS*) substitution (*dN*/*dS*) and can indicate natural selection acting on the proteins. All of the concatenated 13 PCGs (Table S3) were used in the analysis and the values for *ω* ratio >1, =1 or <1 indicate positive selection, neutral evolution or negative selection, respectively (Yang, 2007). To investigate whether positive selection occurred on specific branches, branch models were run under the one-ratio model (M0) or the two-ratio model with *A. americanus* or *Bufotes pewzowi* as the foreground branch, respectively. M0 assumes that all branches have the same *ω* ratio values whereas the two-ratio model assumes one *ω* ratio value for the branches of interest and the other for the background branches. Because different topologies of trees will affect the results, both of the phylogenetic trees structured in BI and ML were used in the analyses. In addition, a likelihood ratio test (LRT) was performed to assess the significant difference between the results of the M0 and the two-ratio model (Yu et al., 2011). Variation happens mostly in several base pairings and may affect a few sites in some lineages (Yang, 2007; Yu et al., 2011). Consequently, the site model was applied to detect the potential selection among sites and allow for different *ω* ratios in different sites, codons or amino acids (Yang, 2007). Seven useful codon substitution models were taken into account in the calculations, including M0 (one ratio), M1a (Nearly Neutral), M2a (Positive Selection), M7 (*β*), M8 (*β* & *ω*) and M8a (*β* & *ω* = 1). We also used LRTs to assess these models and Bayes Empirical Bayes (BEB) to evaluate the posterior probability of positive selection sites. The three-dimensional (3D) structures of the amino acid positive selections in the ND6 protein were formed using SWISS-MODEL Workspace (Waterhouse et al., 2018).

## Results and Discussion

### Mitogenome organization and arrangement

**Figure 1:**
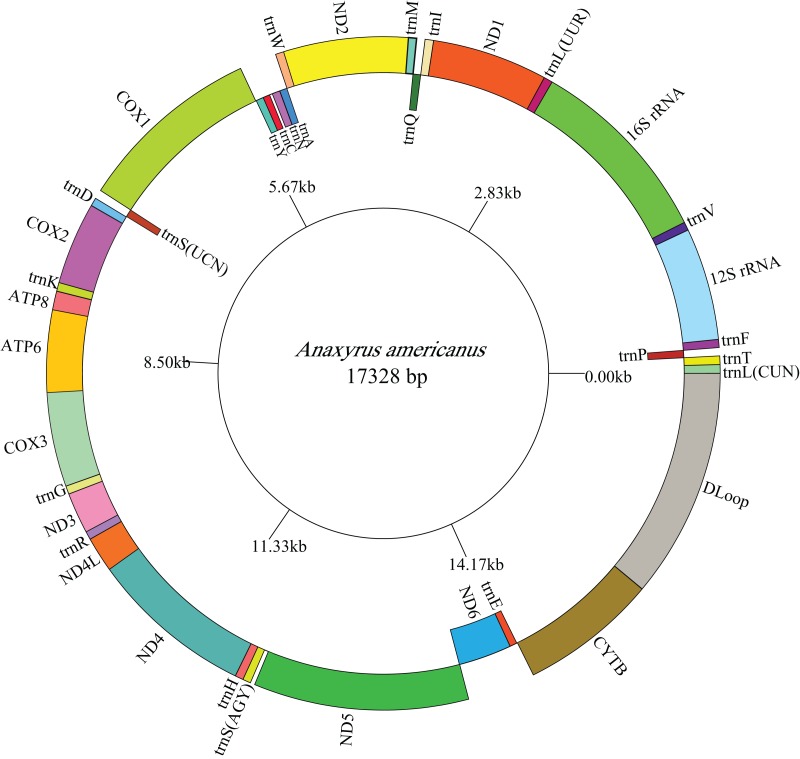
Mitogenome map of *A. americanus*. The one-letter amino acid codes are used to label the tRNAs. Gene names on the inside indicate that the direction of transcription is from left to right, whereas gene names on the outside indicate right to left transcription.

**Figure 2:**
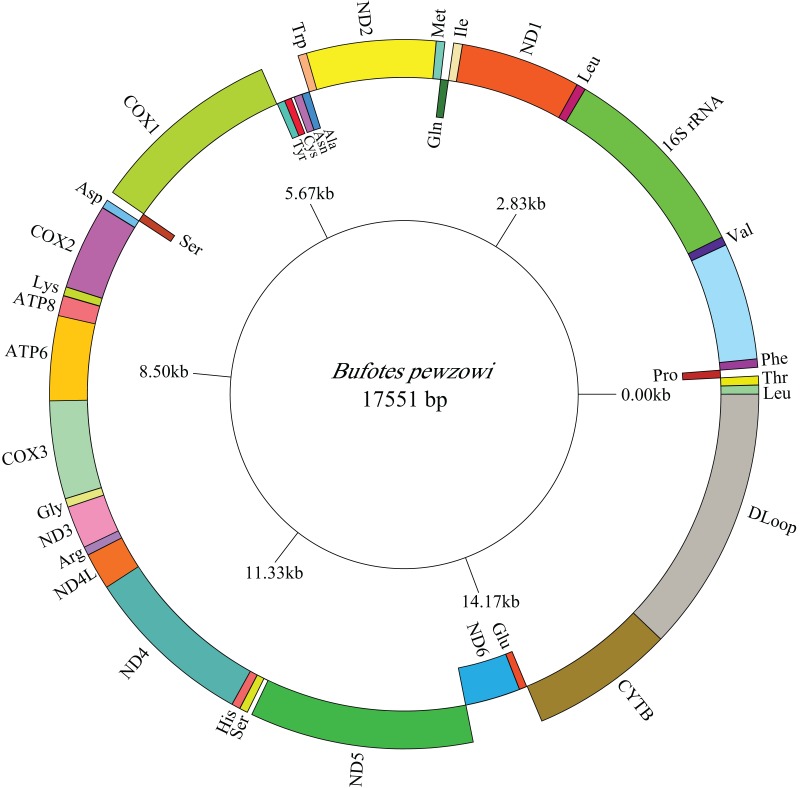
Mitogenome map of *B. pewzowi*. The one-letter amino acid codes are used to label the tRNAs. Gene names on the inside indicate that the direction of transcription is from left to right, whereas gene names on the outside indicate right to left transcription.

The lengths of the complete *A. americanus* and *Bufotes pewzowi* mitogenomes are 17,328 base pairs (bp) and 17,551 bp, respectively (Table 2). Both mitogenomes are circular and contain the typical 37 genes (Tables 3 and 4). Most of the genes are coded on the H-strand, except for 8 tRNA genes and the *ND6* gene on the L-strand. Gene structures are detailed in Figs. 1 and 2. In addition, the gene order and composition are identical with that of other mitogenomes of Bufonidae (Dong & Yang, 2016; Jiang et al., 2017b; Zhang et al., 2016). The different lengths of the mitogenomes are primarily caused by the different sizes of intergenic nucleotides (IGNs) (Tables 3 and 4), particularly the length of the CRs. The overall base composition, A+T and G+C content, as well as AT and GC skew of the *A. americanus* and *Bufotes pewzowi* genomes are listed in Table 2; these data show that *A. americanus* has the highest A+T content (62.4%) and a strong A+T bias. The H-strand of both mitogenomes showed a negative AT-skew and GC-skew, which is expected for most vertebrates (Fonseca, Froufe & Harris, 2006; Hao, Ping & Zhang, 2016; Zhang et al., 2019). Previous studies showed that the asymmetry of the nucleotides resulted primarily from mutations affecting the H-strand during its single-stranded state (Sahyoun et al., 2014).

**Table 2:** The mitogenome composition of *A. americanus* and *Bufotes pewzowi*.

Region	Size (bp)		A+T content		C+G content		AT-skew		CG-skew	
	*A. americanus*	*B. pewzowi*	*A. americanus*	*B. pewzowi*	*A. americanus*	*B. pewzowi*	*A. americanus*	*B. pewzowi*	*A. americanus*	*B. pewzowi*
Whole Genome	17,328	17,551	62.4	59.1	37.6	40.9	−0.038	−0.002	−0.248	−0.271
PCGs	11,290	11,290	61.9	58.6	38	41.5	−0.111	−0.085	−0.256	−0.272
rRNA	2,536	2,543	60.7	58.8	39.4	41.2	0.117	0.149	−0.086	−0.103
tRNA	1,534	1,535	58.9	59.5	41.2	40.4	0.039	0.028	0.014	0.04
CRs	1,916	2,129	70.3	63	29.7	37.1	−0.030	0.006	−0.205	−0.294

**Table 3:** The mitogenome gene characteristics and location of [i] *A. americanus.*

Gene	Strand	Position	Length (nuc.)	Anticodon	Start codon	Stop codon	Intergenic nucleotides
tRNA^Leu^	+	1–72	72	TAG			0
tRNA^Thr^	+	73–144	72	TGT			0
tRNA^Pro^	−	144–212	69	TGG			−1
tRNA^Phe^	+	212–279	68	GAA			−1
12S rRNA	+	280–1213	934				0
tRNA^V al^	+	1214–1282	69	TAC			0
16S rRNA	+	1283–2884	1602				0
tRNA^Leu^	+	2885–2957	73	TAA			0
ND1	+	2958–3918	961		TTG	T	0
tRNA^Ile^	+	3919–3989	71	GAT			0
tRNA^Gln^	−	3989–4059	71	TTG			−1
tRNA^Met^	+	4059–4127	69	CAT			−1
ND2	+	4128–5162	1035		ATT	TAG	0
tRNA^Trp^	+	5161–5230	70	TCA			−2
tRNA^Ala^	−	5232–5300	69	TGC			+1
tRNA^Asn^	−	5302–5374	73	GTT			+1
L-strand origin of replication		5375–5403	29				0
tRNA^Cys^	−	5401–5464	64	GCA			−3
tRNA^Tyr^	−	5465–5534	70	GTA			0
COX1	+	5539–7080	1542		ATA	TAA	+4
tRNA^Ser^	−	7081–7151	71	TGA			0
tRNA^Asp^	+	7156–7224	69	GTC			+4
COX2	+	7226–7910	685		ATG	T	+1
tRNA^Lys^	+	7911–7982	73	TTT			0
ATP8	+	7984–8148	165		ATG	TAA	+1
ATP6	+	8139–8822	684		ATG	TAA	−10
COX3	+	8822–9605	784		ATG	T	−1
tRNA^Gly^	+	9606–9674	69	TCC			0
ND3	+	9675–10016	340		ATG	T	0
tRNA^Arg^	+	10015–10083	69	TCG			0
ND4L	+	10084–10383	300		ATG	TAA	0
ND4	+	10377–11741	1365		ATG	TAA	−7
tRNA^His^	+	11742–11810	69	GTG			0
tRNA^Ser^	+	11811–11877	67	GCT			0
ND5	+	11915–13703	1789		ATG	T	+37
ND6	−	13701–14195	495		ATG	AGA	−3
tRNA^Glu^	−	14196–14263	68	TTC			0
CYTB	+	14269–15411	1143		ATG	AGG	+5
control region		15412–17328	1917				0

**Table 4:** The mitogenome gene characteristics and location of *Bufotes pewzowi*.

Gene	Strand	Position	Length (nuc.)	Anticodon	Start codon	Stop codon	Intergenic nucleotides
tRNA^Leu^	+	1–72	72	TAG			0
tRNA^Thr^	+	73–144	72	TGT			0
tRNA^Pro^	−	144–212	69	TGG			−1
tRNA^Phe^	+	212–279	68	GAA			−1
12S rRNA	+	280–1213	934				0
tRNA^V al^	+	1214–1282	69	TAC			0
16S rRNA	+	1283–2891	1609				0
tRNA^Leu^	+	2892–2964	73	TAA			0
ND1	+	2965–3925	961		TTG	T	0
tRNA^Ile^	+	3926–3996	71	GAT			0
tRNA^Gln^	−	3996–4066	71	TTG			−1
tRNA^Met^	+	4066–4134	69	CAT			−1
ND2	+	4135–5169	1035		ATT	TAG	0
tRNA^Trp^	+	5168–5237	70	TCA			−2
tRNA^Ala^	−	5238–5306	69	TGC			0
tRNA^Asn^	−	5307–5379	73	GTT			0
L-strand origin of replication		5380–5407	28				0
tRNA^Cys^	−	5405–5468	64	GCA			−3
tRNA^Tyr^	−	5469–5538	70	GTA			0
COX1	+	5543–7084	1542		ATA	TAA	+4
tRNA^Ser^	−	7087–7157	71	TGA			+2
tRNA^Asp^	+	7159–7227	69	GTC			+1
COX2	+	7229–7916	688		ATG	T	+1
tRNA^Lys^	+	7917–7988	72	TTT			0
ATP8	+	7990–8154	165		ATG	TAA	+1
ATP6	+	8151–8828	678		ATA	TAA	−4
COX3	+	8828–9611	784		ATG	T	−1
tRNA^Gly^	+	9612–9680	69	TCC			0
ND3	+	9681–10022	342		ATG	TAA	0
tRNA^Arg^	+	10021–10089	69	TCG			−2
ND4L	+	10090–10389	300		ATG	TAA	0
ND4	+	10383–11747	1365		ATG	TAA	−7
tRNA^His^	+	11748–11816	69	GTG			0
tRNA^Ser^	+	11817–11883	67	GCT			0
ND5	+	11923–13711	1789		ATG	T	+39
ND6	−	13709–14203	495		ATG	AGG	−3
tRNA^Glu^	−	14204–14272	69	TTC			0
CYTB	+	14277–15422	1146		ATG	AGA	+4
control region		15423–17551	2129				0

### Protein-coding genes and codon usages

**Figure 3:**
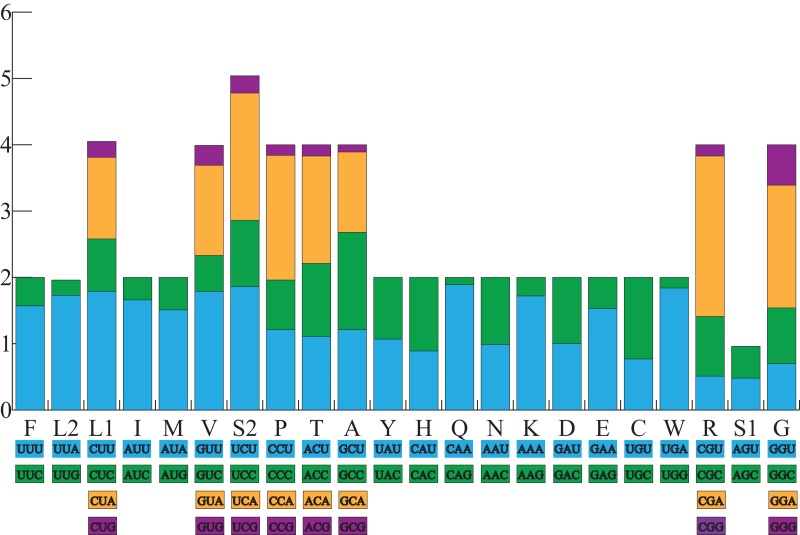
The relative synonymous codon usage (RSCU) in the *A. americanus* mitogenome. All codons used as well as the different combinations of synonymous codons are listed on the x-axis, whereas RSCU values are listed on the *y*-axis. Each amino acid is replaced by one letter, except for Leu and Ser which have two abbreviations because they have two different codons.

**Figure 4:**
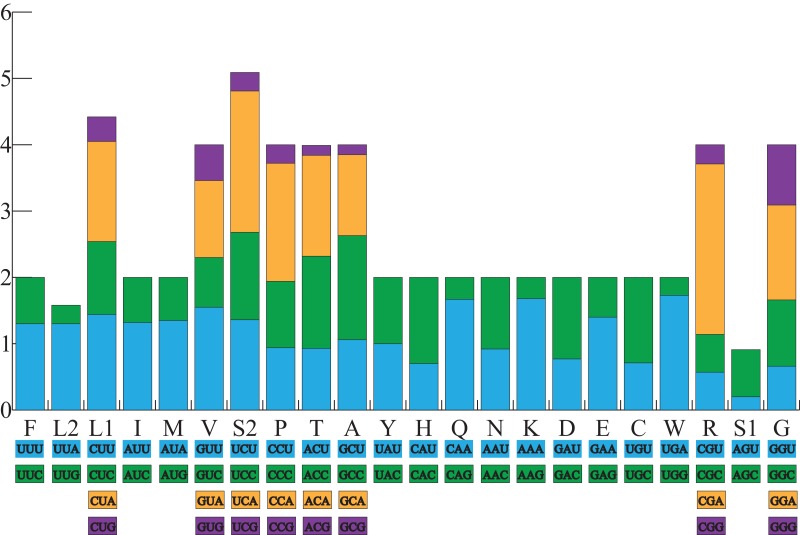
The relative synonymous codon usage (RSCU) in the *B. pewzowi* mitogenome. All codons used as well as the different combinations of synonymous codons are listed on the *x*-axis, whereas RSCU values are listed on the y-axis. Each amino acid is replaced by one letter, except for Leu and Ser which have two abbreviations because they have two different codons.

All the typical 13 PCGs ranged from 165 bp (*ATP8*) to 1,789 bp (*ND5*) in both *A. americanus* and *Bufotes pewzowi*. In addition, the total size of the 13 PCGs in *A. americanus* and *Bufotes pewzowi* were identical (11,290 bp). Mitochondrial PCGs have no introns, but have several overlapping nucleotides with the adjacent gene. There are 5 and 6 reading frame overlaps in the mitogenome of *A. americanus* and *Bufotes pewzowi*, respectively (Tables 3 and 4). All PCGs begin with ATA or ATG as the start codons, except *ND1* that begins with TTG. Although TTG is an uncommon start codon among PCGs, it is often found in amphibians (Wang, Cao & Li, 2017; Zhang et al., 2005). The stop codons for *A. americanus* and *Bufotes pewzowi* are mostly complete TAA, AGG, AGA, and TAG codons with some incomplete T codons. The functionality of these latter is believed to be restored by post-transcriptional polyadenylation (Liu, Wang & Su, 2005; Ojala, Montoya & Attardi, 1981). Furthermore, a comparative analysis indicated that the mitogenome of *A. americanus* had the highest A+T content (61.9%) (Dong & Yang, 2016; Jacob Machado et al., 2018). We also analyzed the relative synonymous codon usage (RSCU) of the *A. americanus* and *Bufotes pewzowi* mitogenomes, excluding stop codons (Table 5; Figs. 3 and 4). The results show that A and T at the third codon position are slightly overused when compared to the synonymous codons of C and G. Moreover, we found that the most frequently used amino acids in these two mitogenomes are *Leu* (CUN), *Ile* and *Ala*, with those encoding *Cys* and *Ser* (AGY) being rare.

**Table 5:** RSCU information for the mitochondrial protein-coding genes of *A. americanus* and *Bufotes pewzowi*.

	*A. americanus*		*B. pewzowi*			*A. americanus*		*B. pewzowi*			*A. americanus*		*B. pewzowi*			*A. americanus*		*B. pewzowi*	
Codon	Count	RSCU	Count	RSCU	Codon	Count	RSCU	Count	RSCU	Codon	Count	RSCU	Count	RSCU	Codon	Count	RSCU	Count	RSCU
UUU(F)	202	1.57	168	1.3	UCU(S2)	93	1.86	67	1.36	UAU(Y)	58	1.07	56	1	UGU(C)	12	0.77	10	0.71
UUC(F)	55	0.43	90	0.7	UCC(S2)	50	1	65	1.32	UAC(Y)	50	0.93	56	1	UGC(C)	19	1.23	18	1.29
UUA(L2)	176	1.73	130	1.3	UCA(S2)	96	1.92	105	2.13	UAA(*)	5	2.5	6	2.67	UGA(W)	103	1.84	96	1.73
UUG(L2)	23	0.23	28	0.28	UCG(S2)	13	0.26	14	0.28	UAG(*)	1	0.5	1	0.44	UGG(W)	9	0.16	15	0.27
CUU(L1)	182	1.79	144	1.44	CCU(P)	61	1.21	47	0.94	CAU(H)	38	0.89	31	0.7	CGU(R)	9	0.51	10	0.57
CUC(L1)	80	0.79	110	1.1	CCC(P)	38	0.75	50	1	CAC(H)	47	1.11	58	1.3	CGC(R)	16	0.9	10	0.57
CUA(L1)	125	1.23	151	1.51	CCA(P)	95	1.88	89	1.78	CAA(Q)	83	1.89	76	1.67	CGA(R)	43	2.42	45	2.57
CUG(L1)	24	0.24	37	0.37	CCG(P)	8	0.16	14	0.28	CAG(Q)	5	0.11	15	0.33	CGG(R)	3	0.17	5	0.29
AUU(I)	281	1.66	214	1.32	ACU(T)	78	1.11	66	0.93	AAU(N)	67	0.99	61	0.92	AGU(S1)	24	0.48	10	0.2
AUC(I)	57	0.34	111	0.68	ACC(T)	77	1.1	99	1.39	AAC(N)	68	1.01	72	1.08	AGC(S1)	24	0.48	35	0.71
AUA(M)	131	1.51	116	1.35	ACA(T)	114	1.62	108	1.52	AAA(K)	73	1.72	74	1.68	AGA(*)	1	0.5	1	0.44
AUG(M)	42	0.49	56	0.65	ACG(T)	12	0.17	11	0.15	AAG(K)	12	0.28	14	0.32	AGG(*)	1	0.5	1	0.44
GUU(V)	83	1.79	75	1.55	GCU(A)	92	1.21	83	1.06	GAU(D)	37	1	29	0.77	GGU(G)	39	0.7	37	0.66
GUC(V)	25	0.54	36	0.75	GCC(A)	111	1.47	123	1.57	GAC(D)	37	1	46	1.23	GGC(G)	47	0.84	56	1
GUA(V)	63	1.36	56	1.16	GCA(A)	92	1.21	96	1.22	GAA(E)	71	1.53	63	1.4	GGA(G)	103	1.85	80	1.43
GUG(V)	14	0.3	26	0.54	GCG(A)	8	0.11	12	0.15	GAG(E)	22	0.47	27	0.6	GGG(G)	34	0.61	51	0.91

### Ribosomal and transfer RNA genes

We found that the length of rRNAs in *Bufotes pewzowi* (2,543 bp) is longer than other Bufonidae species (Cao et al., 2006; Igawa et al., 2008; Jiang et al., 2017b; Wang et al., 2013; Yang et al., 2016). The complete mitogenome of *A. americanus* or *Bufotes pewzowi* contains 22 typical tRNA genes, which ranged from 64 bp to 73 bp in length. In the H-strand, *A. americanus* showed a positive AT-skew and GC-skew of the tRNAs, and *Bufotes pewzowi* also showed a positive AT-skew and GC-skew (Table 2). Furthermore, all of the tRNA genes displayed the potential to fold into the typical cloverleaf secondary structure, excluding the *trnC* and *trnS* (AGY) (Figs. S1 and S2). In both species, the *trnS* (AGY) has an extra unpaired adenine (A) in the anticodon arm and the *trnC* has lost the DHU loop. This phenomenon is also found in other Bufonidae species (shown in Fig. S3) (Cao et al., 2006; Igawa et al., 2008; Zhang et al., 2005; Zhang et al., 2013). The *trnS* (AGY) in *A. americanus* had also lost the DHU arm, whereas in *Bufotes pewzowi* lost only the DHU loop. Hence, the unusual secondary structure of *trnS* (AGY) is in line with the molecular trend observed in metazoa (Wolstenholme, 1992). We also found 13 unmatched base pairs in *A. americanus* and 15 in *Bufotes pewzowi*. The putative origin of L-strand replications (O_L_) are 29 bp and 28 bp long in *A. americanus* and *Bufotes pewzowi*, respectively. Both of these have the potential to fold into the characteristic stem and loop structure (Jiang et al., 2017a; Sahyoun et al., 2014) that has been demonstrated by Hixson & Brown (1986) to be involved in the transition from RNA to DNA synthesis.

### Control region and intergenic regions

The control regions (CRs) in *A. americanus* and *Bufotes pewzowi* have lengths of 1,916 bp and 2,129 bp, respectively. The A+T content value (70.3%) of *A. americanus* is the highest known to date among Bufonidae species. In addition, *A. americanus* showed a negative AT-skew and GC-skew in its CR, whereas *Bufotes pewzowi* showed a positive AT-skew and negative GC-skew. Guanine (G) was the scarcest nucleotide at the third codon position of the H-strand due to a strong bias against guanine usage in the *A. americanus* mitogenome, which is common for mitogenome strands of vertebrates (Kan et al., 2010). The CRs can be divided into three regions that depend on the distribution of the variable nucleotide positions and differential frequencies of the nucleotides, and contain repeat regions at both 5′ and 3′-sides. We found 3 tandem repeats in *A. americanus* with consensus sizes of 162 bp, 21 bp and 2 bp. In *Bufotes pewzowi* there are only two tandem repeats whose consensus sizes were 104 bp and 91 bp. The intergenic spacers (IGNs) have a variable length within the mitogenomes of *A. americanus* (Table 3) and *Bufotes pewzowi* (Table 4). All lengths of IGNs in both species are smaller than 5 bp, except for one that was 37 bp in *A. americanus* and 39 bp in *B. pewzowi*, both located between the *trnS* (AGY) and *ND5* genes. Comparative analysis showed that the longest IGN between *A. americanus* and *Bufotes pewzowi* had a high similarity (76.9%). In many vertebrates, there is usually a *trnL* (CUN) gene following the *trnS* (AGY) gene (Cheng et al., 2018; Lin et al., 2014; Ni et al., 2016; Ye et al., 2016; Yu et al., 2012; Yu, Zhang & Zheng, 2012). However, when the *trnL* (CUN) gene is translocated, an IGN is left in its original position (Zhou et al., 2009). Thus, Cao et al. (2006) speculated that this might reflect the evolution of the mitogenome arrangement in Anura.

**Figure 5:**
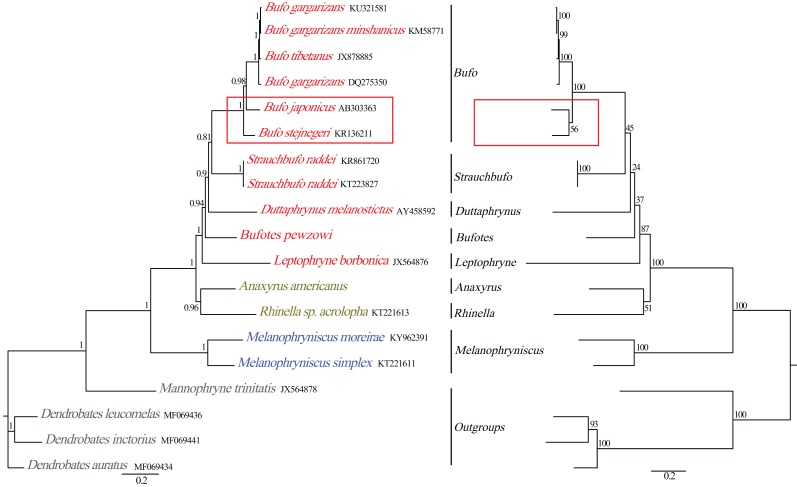
Phylogenetic relationships among Bufonidae in BI and ML analyses. The BI analysis result is shown on the left and the numbers above the nodes are posterior probability. The ML analysis is on the right and the numbers above the nodes are bootstrap value.

### Phylogenetic analyses

Phylogenetic relationships based on the nucleotide sequences of the 13 PCGs were obtained with BI and ML analyses (Fig. 5). Our BI analysis was relatively robust and provided resolution with high clade posterior probabilities, whereas the ML results showed some modest clade frequencies. Furthermore, the phylogenetic relationships deduced from BI and ML analyses showed somewhat different topologies. In the BI and ML analyses, the monophyly of Bufonidae was well recovered. In the BI analysis, *Bufo japonicus* was a sister group to the clade of (*Bufo gargarizan* DQ275350 + (*Bufo tibetanus* JX878885 + (*Bufo gargarizan* KM587710 + *Bufo gargarizan* KU321581))). However, in the ML analysis, *Bufo japonicus* was a sister group to *Bufo stejnegeri* and then the group of (*Bufo japonicus* + *Bufo stejnegeri*) was a sister group to the cluster of (*Bufo gargarizan* DQ275350 + (*Bufo tibetanus* JX878885 + (*Bufo gargarizan* KM587710 + *Bufo gargarizan* KU321581))). Coincidentally, Dong & Yang (2016) also reported that *Bufo japonicus* was a sister group to *Bufo stejnegeri* and the monophyly of family Bufonidae and genus *Bufo* were corroborated. Frost et al. (2006) and Van Bocxlaer et al. (2009) corroborated the monophyly of Bufonidae as well as the results of Portik & Papenfuss (2015). Our phylogeny within Bufonidae is generally similar to the results of Pramuk et al. (2008), Ron et al. (2015) and Dong & Yang (2016). The ML and BI analyses also confirmed the monophyly of *Bufo*, which has also been demonstrated by other researchers (Graybeal, 1997; Dong & Yang, 2016). By contrast, our results were different from Brandvain et al. (2014) and Jiang et al. (2018), who reported that *Bufo* was a paraphyletic group, a result that was also deduced by Pyron & Wiens (2011). However, we found that Dong & Yang (2016) confirmed the monophyly of *Bufo* using the complete mitogenomes, whereas Brandvain et al. (2014), Pyron & Wiens (2011) and Jiang et al. (2018) confirmed a paraphyletic relationship using partial mitochondrial genes (*12S*, *16S* and *CYTB*) and several nuclear genes. Hence, the different results from these studies are likely caused by the different data sets and/or methods applied.

The family Bufonidae was divided into 3 major groups: (*Leptophryne* + (*Bufotes* + (*Duttaphrynus* + (*Strauchbufo* + (genus *Bufo*))))), (*A. americanus* + *Rhinella acrolopha*), and (*Melanophryniscus simplex* + *M. moreirae*). Among them, the clade of *A. americanus* was the sister group to *R. acrolopha* whereas *Bufotes pewzowi* was a sister group to the clade of (*Duttaphrynus* + (*Strauchbufo* + (genus *Bufo*))). Genus *Anaxyrus* was a sister group to genus *Rhinella* and then the clade of (*A. americanus* + *R. acrolopha*) was the sister group to (*Leptophryne* + (*Bufotes* + (*Duttaphrynus* + (*Strauchbufo* + (*Bufo*))))). Portik & Papenfuss (2015) also found that genus *Anaxyrus* was the sister group to genus *Rhinella*. Our phylogenetic tree showed that the clade of (*M. simplex* + *M. moreirae*) (BI: 1, ML: 100%) was recovered as the sister group to the other Bufonidae species. We also found a similar result in the research of Portik & Papenfuss (2015). The South American genera include *Melanophryniscus*, *Nannophryne*, as well as the group of *Rhinella* that is endemic to South America and is distributed from the southern tip of Texas to as far south as Argentina (Vallinoto et al., 2010). This phenomenon was consistent with the results from other researchers and implies that Bufonidae may have originated from South America. Coincidentally, nearly all recent phylogenetic analyses have asserted a South American origin for Bufonidae (Pérez-Ben, Gómez & Báez, 2019; Pramuk et al., 2008). In addition, discrepancies in the results between our work and prior studies were likely to be caused by the different composition of the data chosen (Gao et al., 2018). Therefore, more molecular data are needed to better comprehend the phylogenetic relationship of the family, especially from taxa that, in other studies, led to a conclusion that *Bufo* was paraphyletic.

**Table 6:** Summary of EasyCodeML analysis of positive selection on mitogenomes based on the phylogenetic tree from BI.

Foreground branch	Models	ℓn L	Estimates of parameters	Model compared	LRT *P*-value	Positive sites
Branch model						
*A. americanus*	M0	−64953.9215	*ω* = 0.03375	M0 vs. two-ratios model	0.000000011	
	two-ratios model	−64937.6024	*ω*0 = 0.03520, *ω*1 = 0.01846			
*Bufotes pewzowi*	M0	−64953.9215	*ω* = 0.03375	M0 vs. two-ratios model	0.588391030	
	two-ratios model	−64953.7750	*ω*0 = 0.03390, *ω*1 = 0.03200			
Site model						
	M0	−64953.9215	*ω*0 = 0.03375	M0 vs. M3	0.000000000	[] Not Allowed
	M3	−63675.0885	*ω*0 = 0.00589, *ω*1 = 0.1673, *ω*2 = 101.4548						
			p0=0.8120, p1=0.1880, p2=0.00000			
	M1a	−64441.3475	*ω*0 = 0.02382, *ω*1 = 1.00000	M1a vs. M2a	0.999999000	[] Not Allowed
			p0=0.9533, p1=0.04670						
	M2a	−64441.3475	*ω*0 = 0.02382, *ω*1 = 1.0000, *ω*2 = 40.4741						
			p0=0.9533, p1=0.04670, p2=0.0000			
	M7	−63769.6465	*p* = 0.1688, *q* = 2.4104	M7 vs.M8	0.000000000	3584 I 0.655, 3585 T 0.654, 3590 G 0.925, 3594 D 0.942 Not Allowed
	M8	−63735.9750	*ω*0 = 1.00000, *q* = 2.5906						
			p0=0.9893, p1=0.01066, p2=0.1583			
	M8a	−63639.2914	*ω*0 = 1.00000, *q* = 3.8610	M8a vs.M8	0.000000000	Not Allowed
			p0=0.9985, p1=0.00149, p2=0.1657			

**Table 7:** Summary of EasyCodeML analysis of positive selection on mitogenomes based on the maximum-likelihood tree.

Foreground branch	Models	ℓn L	Estimates of parameters	Model compared	LRT *P*-value	Positive sites
Branch model						
*A. americanus*	M0	−64946.8069	*ω* = 0.03373	M0 vs. two-ratios model	0.000000013	
	two-ratios model	−64930.6151	*ω*0 = 0.03518, *ω*1 = 0.01848			
*Bufotes pewzowi*	M0	−64946.8069	*ω* = 0.03373	M0 vs. two-ratios model	0.531545543	
	two-ratios model	−64946.6112	*ω*0 = 0.03390, *ω*1 = 0.03171			
Site model						
	M0	−64946.8069	*ω*0 = 0.03373	M0 vs. M3	0.000000000	[] Not Allowed
	M3	−63668.4498	*ω*0 = 0.00589, *ω*1 = 0.1674, *ω*2 = 112.9303						
			p0=0.8121, p1=0.1879, p2=0.00000			
	M1a	−64433.7560	*ω*0 = 0.02376, *ω*1 = 1.00000	M1a vs. M2a	0.999941002	[] Not Allowed
			p0=0.9531, p1=0.04689						
	M2a	−64433.7561	*ω*0 = 0.02376, *ω*1 = 1.0000, *ω*2 = 52.2715						
			p0=0.9531, p1=0.04689, p2=0.0000			
	M7	−63763.2701	*p* = 0.1689, *q* = 2.4119	M7 vs.M8	0.000000000	3584 I 0.654, 3585 T 0.655, 3590 G 0.925, 3594 D 0.942 Not Allowed
	M8	−63730.7785	*ω*0 = 1.00000, *q* = 2.5907						
			p0=0.9894, p1=0.01056, p2=0.1586			
	M8a	−63633.2296	*ω*0 = 1.00000, *q* = 3.8578	M8a vs.M8	0.000000000	Not Allowed
			p0=0.9986, p1=0.00145, p2=0.1656			

### Analysis of positive selection in 13 protein-coding genes

The results of positive selection are shown in Tables 6 and 7. In the analyses of the branch model, we reached a similar conclusion no matter which toad species was set as the foreground branch. The *ω* ratio value in the M0 model was 0.03375 when using the BI tree and 0.03373 using the ML tree, with *ω* ratio values all smaller than 1. This means that these two branches are under negative selection. When we set *A. americanus* as the foreground branch, the LRT of the comparison (M0 vs. the two-ratio model) was highly significant (*p* < 0.01), whereas when *Bufotes pewzowi* was set as the foreground branch, the LRT value was greater than 0.05. The site model was used to detect positive selection sites and we got similar conclusions regardless of which tree-building method was used. The LRT of M7-M8 comparison showed high significance (*P* < 0.01) and two amino acid positions were found have BEB values >0.90 (positions 3,590 and 3,594 in the concatenated amino acids of the 13 PCGs). Amino acid residues 3,590 and 3,594 correspond to amino acid positions 115 and 119 in the ND6 protein, respectively (Table S3). Residue glycine (G, 115) in the ND6 protein can be found in *A. americanus*, *Bufo gargarizan*, *Bufo japonicus*, *Bufo stejnegeri*, *Bufo tibetanus* and *S. raddei*, which are distributed across northern regions of the earth at similar latitudes (33°N to 44°N), and residues serine as well as glutamic acid can also be found in this position in other Bufonidae. Residue aspartic acid (D, 119) in the ND6 protein can only be found in *A. americanus* whereas most of the other Bufonidae species contain glycine at this site. Amino acid positions 115 and 119 (Fig. S4) are in a part of the ND6 protein that protrudes outside the mitochondrial inner membrane into the intermembrane space (as determined using the TMHMM Server v. 2.0; http://www.cbs.dtu.dk/services/TMHMM/) (Möller, Croning & Apweiler, 2001). In this study, *A. americanus* was obtained from the highest latitude and contained both of the two positive selection sites. At 115 and 119 positions of ND6 protein, aspartic acid has a negatively charged polar side chain, whereas glycine has an uncharged side chain with no polar group (Shiraishi & Kuwabara, 1970). The amino acid changes in the ND6 protein may have a role in modulating Complex I redox potential and ROS production (Zhuang & Cheng, 2010).

Consequently, the above evidence possibly implies that the mitogenomes of these northern species are under natural selection. The changes in the relative mass and electrical charges of amino acids that they encode may be related to adaptive evolution to low temperature environments in *A. americanus*. As the first enzyme complex of the respiratory chain, mitochondrial complex I consists of 45 subunits, its seven hydrophobic subunits being encoded by the mitogenome (*ND1-6* and *4L*) (Formosa et al., 2018; Zhou et al., 2014). Thus, the adaptive changes in several *ND4* and *ND6* gene sites may affect the proton-pumping process and metabolic performance (Yu et al., 2011). Sun et al. (2018a) found positive selection on *ND4* from *Tetranychus truncatus* during adaptation to low temperature. Zhuang & Cheng (2010) proposed that the modification of *ND6* gene probably improved complex I subunit interactions at low temperatures after analyzing positive selection results. Lamb et al. (2018) also found positive selection of the *ND6* gene when tested for evolution caused by climate-linked selection. Furthermore, residue aspartic acid (D, 119) can also be found in the same position in the ND6 proteins of other anurans, such as in the family Ranidae (*Rana dybowskii*, *R. cf. chensinensis* and *R. huanrenensis*), Leptodactylidae (*Leptodactylus melanonotus*) and Microhylidae (*Phrynomantis microps*). We observe that the three frogs in the family Ranidae are mainly distributed in northern China (Li, Lei & Fu, 2014; Dong, Zhou & Yang, 2015), where temperatures are relatively low. *L. melanonotus* and *P. microps* are distributed near the equator, where temperatures are relatively high. More research is needed to reach definitive conclusions about low or high temperature positive selections in mitochondrial genes (e.g., Banguera-Hinestroza et al., 2018; Ben Slimen et al., 2018; Sun et al., 2018b; Zhou et al., 2014). According to this evidence, we can hypothesize that residue aspartic acid (D, 119) in the ND6 protein of *A. americanus* may be related to temperature adjustment and in different anurans may be under different climate-linked selection. However, we did not find evidence for positive selection in the mitogenome of *Bufotes pewzowi* and this suggests that gene adaptations that improve osmoregulation (if they occur) may be associated with nuclear genes. Hence, more information on the evolution of nuclear genomes in toads is needed to analyze the potential adaptation to salinity.

## Conclusions

The complete mitochondrial genomes of *A. americanus* and *Bufotes pewzowi* were successfully sequenced and annotated. Both show the same gene orders and orientation as occurs in other mitogenomes of Bufonidae, whereas the A+T content of the whole mitogenome in *A. americanus* is the highest among the known species of Bufonidae. It is noteworthy that the *ND1* gene begins with TTG as the start codon and the *trnC* and *trnS* (AGY) genes could not fold into the typical cloverleaf secondary structure in these two toad species, which is a common phenomenon in Bufonidae. Both BI and ML analyses indicated Bufonidae and *Bufo* as monophyletic groups in this study.

Furthermore, foreground branches (*A. americanus* and *Bufotes pewzowi*) are subject to negative selection ( *ω* <1). In the site model, two positive selection sites with BEB values >0.90 were found and both were located in the *ND6* gene. The residue G (115) in ND6 protein can be found in toad species living in northern regions, but residue D (119) in ND6 protein can only be found in *A. americanus*. No positive selection site was found in *Bufotes pewzowi*. The results show that adaptation to low temperature in *A. americanus* may be partly related to evolutionary changes in the *ND6* gene and the residue D (119) in ND6 protein may be linked to temperature adjustment. However, adaptation to high salinity by *Bufotes pewzowi* could not be linked to a modification of its mitogenome.

##  Supplemental Information

10.7717/peerj.8901/supp-1 Supplemental Information 1 The sequence of mitochondrial genome of Bufotes pewzowi

10.7717/peerj.8901/supp-2 Supplemental Information 2 The sequence of mitochondrial genome of Anaxyrus americanus

10.7717/peerj.8901/supp-3Figure S1Inferred secondary structures for the 22 tRNAs of *A.americanus*(A)* trnL* (CUN); (B) * trnT* ; (C) * trnP* ; (D) * trnF* ; (E) * trnV* ; (F) * trnL* (UUR); (G) * trnI* ; (H) * trnQ* ; (I) * trnM* ; (J) * trnW* ; (K) * trnA* ; (L) * trnN* ; (M) * trnC* ; (N) * trnY* ; (O) * trnS* (UCN); (P) * trnD* ; (Q) * trnK* ; (R) * trnG* ; (S) * trnR* ; (T) * trnH* ; (U) * trnS* (AGY); (V) * trnE* . $\hat {}$ Arms of the tRNAs (clockwise from the top) are the amino acid acceptor arm, the T *ψ* C arm, the anticodon arm, and the DHU arm.

10.7717/peerj.8901/supp-4 Figure S2 Inferred secondary structures for the 22 tRNAs of *Bufotespewzowi* (A) * trn L* (CUN); (B) * trnT* ; (C) * trnP* ; (D) * trnF* ; (E) * trnV* ; (F) * trnL* (UUR); (G) * trnI* ; (H) * trnQ* ; (I) * trnM* ; (J) * trnW* ; (K) * trnA* ; (L) * trnN* ; (M) * trnC* ; (N) * trnY* ; (O) * trnS* (UCN); (P) * trnD* ; (Q) * trnK* ; (R) * trnG* ; (S) * trnR* ; (T) * trnH* ; (U) * trnS* (AGY); (V) * trnE*. Arms of the tRNAs (clockwise from the top) are the amino acid acceptor arm, the T *ψ*C arm, the anticodon arm, and the DHU arm.

10.7717/peerj.8901/supp-5 Figure S3 Inferred secondary structures for *trnC* of all available Bufonidae species Arms of tRNAs (clockwise from the top) are the amino acid acceptor arm, the T*ψ* C arm, the anticodon arm, and the DHU arm.

10.7717/peerj.8901/supp-6 Figure S4 Three-dimensional representation of relevant variable amino acids in the *A. americanus ND6* gene Mutations in sites shown with black number have been related to low temperature tolerance in *A. americanus*.

10.7717/peerj.8901/supp-7 Table S1 Universal primers used in this study

10.7717/peerj.8901/supp-8 Table S2 Specific primers used in this study

10.7717/peerj.8901/supp-9 Table S3 The order and length of nucleotide and amino acid from 13 protein-coding genes were used in the analysis

## Additional Information and Declarations

### Field Study Permissions

The following information was supplied relating to field study approvals (i.e., approving body and any reference numbers): Ontario Ministry of Natural Resources, Wildlife Scientific Collector’s Authorization, #1082196, was issued February 1, 2016, expiry date December 31, 2016, to Dr. Kenneth Storey, Department of Biology, Carleton University.

### DNA Deposition

The following information was supplied regarding the deposition of DNA sequences: The complete mitochondrial genome sequences are available at GenBank: MK855099 and MK855100.

### Data Availability

The following information was supplied regarding data availability: The raw measurements are available in the Supplementary Files.
