# Supplementary figures and images for: Characterization of the mitochondrial genomes of two toads, Anaxyrus americanus (Anura: Bufonidae) and Bufotes pewzowi (Anura: Bufonidae), with phylogenetic and selection pressure analyses
Source: PeerJ. 2020 Apr 14;8:e8901. doi: 10.7717/peerj.8901 (PMC7164433; doi:10.7717/peerj.8901)

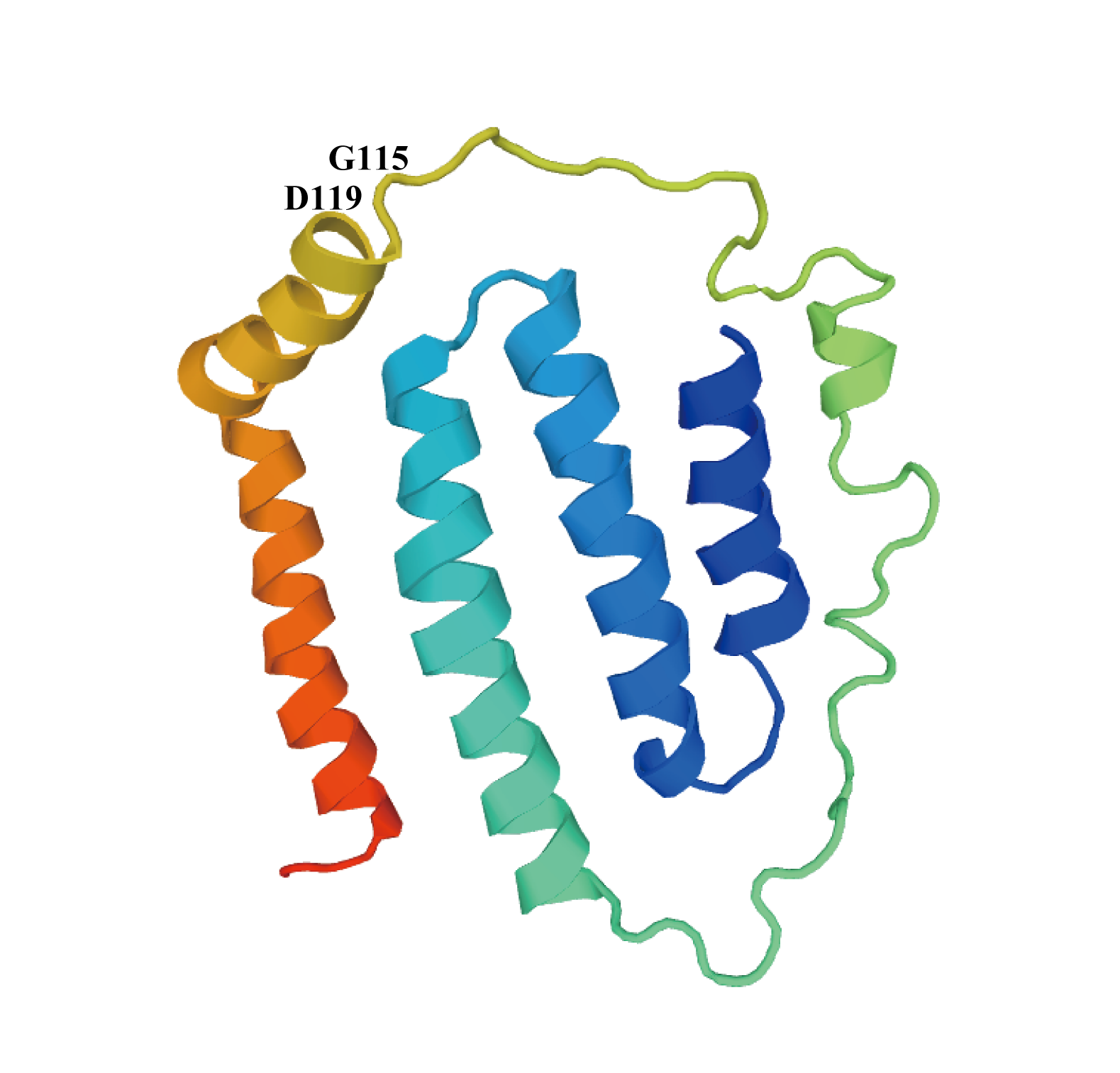

Supplement: Figure S4 — Mutations in sites shown with black number have been related to low temperature tolerance in A. americanus. [file peerj-08-8901-s006.png]
