# Supplementary material for: Characterization of the mitochondrial genomes of two toads, Anaxyrus americanus (Anura: Bufonidae) and Bufotes pewzowi (Anura: Bufonidae), with phylogenetic and selection pressure analyses
Source: PeerJ. 2020 Apr 14;8:e8901. doi: 10.7717/peerj.8901 (PMC7164433; doi:10.7717/peerj.8901)
Supplement: Table S1 [file peerj-08-8901-s007.doc]

Table S1

| Fragment | Primer name | Primer sequences (5’ to 3’) |
| --- | --- | --- |
| U1 | NFlle3700L | GAAAGHHARGGNYCTCCTTGATAG |
|  | FAsn5150H | AAGTAGAAWGAAGCTCGCYGG |
| U2 | ZW-16S-2U | ACCCCTCGTACCTYTTGCATCATG |
|  | ZW-16STY-1D | CTCCGGTCTGAACTCAGATCACGTAGG |
| U3 | 12SALa | AAACTGGGATYAGATACCCCACTAT |
|  | 16S2000Ha | GTKATTAYGCTACCTTYGCACGGT |
| U4 | LX12SN1a | GACRTGTACACACCGCCCGTC |
|  | lX16S1Ra | GACCTGGATTRCTCCGGTCTGAACTC |
| U5 | LX16S1a | GGTTTACGACCTCGATGTTGGATCA |
|  | Met3850Ha | GGTATGGGCCCAARAGCTT |
| U6 | FTrp5000L | AGACCWARRGCCTTCAAAGY |
|  | FCOll7050H | ATAATHGGNGADRYTGCRTCTTG |
| U7 | FSer6900L | CGAGAAARGRRGGAATYGAAC |
|  | FCOlll8650H | GGTCADGGRYTDGGGTCWACTAT |
| U8 | FLys7750L | AGCGWCARCCTTTTAAGYT |
|  | FArg9840H | TAAGYCGAAATYARYTRTCTT |
| U9 | FHis11600L | ARAAYWYTAGATTGTGATTCTA |
|  | FND512800H | CCTATTTTDCGRATRTCYTGYTC |
| U10 | PFGlu14140L | GAAAAACCACTGTTGTHHYTCAACTA |
|  | PFThr15310H | CGGYTTACAAGACCRRTGCTTT |
| U11 | FPhe50L | CTGAARAYGCTRAGATGRRCCCTRAAAAG |
|  | 12S600Ha | TTATCGATKATRGRACAGGCTCCTCT |

Note: universal primer is replaced by a single letter U
