# Supplementary material for: Characterization of the mitochondrial genomes of two toads, Anaxyrus americanus (Anura: Bufonidae) and Bufotes pewzowi (Anura: Bufonidae), with phylogenetic and selection pressure analyses
Source: PeerJ. 2020 Apr 14;8:e8901. doi: 10.7717/peerj.8901 (PMC7164433; doi:10.7717/peerj.8901)
Supplement: Table S2 [file peerj-08-8901-s008.doc]

**Table S2**

| Fragment | Primer name | Primer sequences (5’ to 3’) |
| --- | --- | --- |
| SA1 | BMCC-J-450 | GCAGTGATTAACATTGAGCA |
|  | BMCC-N-1845 | GAAGCCAGTATATCCATTAGTC |
| SA2 | BMCC-J-5789 | CCTCATTCCTCCTTCTTCTG |
|  | BMCC-N-6811 | TCGTGGTATTCCTGCTAATC |
| SA3 | BMCC-J-9661 | GACTCCCTTACTCAATACGA |
|  | BMCC-N-12357 | TAATACCTACTCCTTCTCATCC |
| SA4 | BMCC-J-FArg | TATCAAGGACAACCAAACTG |
|  | BMCC-N-FHis | AGAAAAAGTCCAGTTGC |
| SA5 | BMCC-Glu14140L | TAACCTRGACTTAYAGYYTGAAAA |
|  | BMCC-CB15160H | TCTTCDACTGGYTGBCCBCCRAT |
| SA6 | BMCC-J-CR1 | GGAACTGAATCTATGGACTT |
|  | BMCC-N-CR1 | AGTAATAACTGACCTCAAGG |
| SA7 | BMCC-J-10/6 | CAACTTCACACCAGCCAATCCTCTT |
|  | BMCC-N-10/6 | GGTATCTAATCCCAGTTTGTTCCCTAGC |
| SB1 | TLMCC-J-6851 | TATTCACGGTGGGAGGGTTG |
|  | TLMCC-N-8526 | TAGGGCTATTGTGGCGGTTG |
| SB2 | TLMCC-J-7026 | TGATTCTGACCGTGACCATA |
|  | TLMCC-N-8693 | AAAGGATAGCCACAAGGAAA |
| SB3 | TLMCC-J-9577 | ACTACCAACAATAAACTCAGACT |
|  | TLMCC-N-10312 | GGTGAAATGATAAGCCAATA |
| SB4 | TLMCC-J-9742 | CTGGGCTTTATCTACGAATG |
|  | TLMCC-N-12236 | ATGGCAAATACTCAAGGTGT |
| SB5 | TLMCC-J-12236 | TCTAGCACAATAGTAGTAGCC |
|  | TLMCC-N-13864 | TACCAGGAAGAGAAGAATCAC |
| SB6 | TLMCC-J-15098 | AACACGATTCTTCACATTCC |
|  | TLMCC-N-17983 | TAACTGACTTCAAGGCTAGG |
| SB7 | TLMCC-Glu14140L | TAACCTAGACYCACAGTCYGAAAA |
|  | TLMCC-CB15160H | TCYTCTACTGGTTGNCCYCCRAT |
| SB8 | TLMCC-J-Cb12S | AGAGCATACACCTAACTTCC |
|  | TLMCC-N- Cb12S | TAACTGACTTCAAGGCTAGG |

Note: specific primers for *A. americanus* are identified as SA and specific primers for *B. pewzowi* as SB.
