# Supplementary material for: Characterization of the mitochondrial genomes of two toads, Anaxyrus americanus (Anura: Bufonidae) and Bufotes pewzowi (Anura: Bufonidae), with phylogenetic and selection pressure analyses
Source: PeerJ. 2020 Apr 14;8:e8901. doi: 10.7717/peerj.8901 (PMC7164433; doi:10.7717/peerj.8901)
Supplement: Table S3 [file peerj-08-8901-s009.doc]

**Table S3 The order and length of nucleotide and amino acid from 13 protein-coding genes were used in the analysis.**

| Gene | Nucleotide position | Length of nucleotide sequence | Amino acid position | Length of amino acid sequence |
| --- | --- | --- | --- | --- |
| *ATP6* | 1-675 | 675 bp | 1-225 | 225 |
| *ATP8* | 676-837 | 162 bp | 226-279 | 54 |
| *COX1* | 838-2,367 | 1,530 bp | 280-789 | 510 |
| *COX2* | 2,368-3,051 | 684 bp | 790-1,017 | 228 |
| *COX3* | 3,052-3,834 | 783 bp | 1,018-1,278 | 261 |
| *CYTB* | 3,835-4,803 | 969 bp | 1,279-1,601 | 323 |
| *ND1* | 4,804-5,739 | 936 bp | 1,602-1,913 | 312 |
| *ND2* | 5,740-6,741 | 1,002 bp | 1,914-2,247 | 334 |
| *ND3* | 6,742-7,080 | 339 bp | 2,248-2,360 | 113 |
| *ND4L* | 7,081-7,377 | 297 bp | 2,361-2,459 | 99 |
| *ND4* | 7,378-8,733 | 1,356 bp | 2,460-2,911 | 452 |
| *ND5* | 8,734-10,428 | 1,695 bp | 2,912-3,476 | 565 |
| *ND6* | 10,429-10,917 | 489 bp | 3,477-3,639 | 163 |
